# Study on Adhesion Property and Moisture Effect between SBS Modified Asphalt Binder and Aggregate Using Molecular Dynamics Simulation

**DOI:** 10.3390/ma15196912

**Published:** 2022-10-05

**Authors:** Fucheng Guo, Jianzhong Pei, Jiupeng Zhang, Rui Li, Pengfei Liu, Di Wang

**Affiliations:** 1 Key Laboratory for Special Area Highway Engineering of Ministry of Education, Chang’an University, Xi’an 710064, China; 2 Institute of Highway Engineering, RWTH Aachen University, Mies-van-der-Rohe-Str. 1, 52074 Aachen, Germany; 3 Department of Civil Engineering, Aalto University, Rakentajanaukio 4, 02150 Espoo, Finland; 4 Hangzhou Telujie Transportation Technology Co., Ltd., Hangzhou 311121, China

**Keywords:** SBS modified asphalt binder, aggregate, interface, adhesion property, moisture effect, molecular dynamics simulation

## Abstract

In this project, the adhesion property and moisture effect between styrene–butadiene–styrene (SBS) modified asphalt binder and aggregate were studied to reveal their interface adhesion mechanism. The influence of SBS contents on adhesion property and moisture effect between binder and aggregate phases were investigated using molecular dynamics simulation. Moreover, the double-layer adhesion models of asphalt binder–aggregate and triple-layer debonding models of asphalt binder–water–aggregate were constructed and equilibrated, and the adhesion property and the moisture effect were evaluated numerically. The results indicate that the built SBS-modified asphalt binder models show favorable reliability in representing the real one. The variation in the work of adhesion for SBS modified asphalt binder–quartz is not remarkable with the SBS content when its content is relatively low. However, the work of adhesion decreased significantly when the content was higher than 6 wt.%, which is consistent with the experimental results. The adhesion between SBS-modified asphalt binder and quartz is derived from Van der Waals energy. The modified asphalt binder with a high SBS modifier content (8 wt.% and 10 wt.%) shows much better moisture resistance (nearly 30% improved) than the unmodified asphalt binder from the work of debonding results. According to the Energy Ratio (*ER*) values, asphalt binders with high SBS content (8 wt.% and 10 wt.%) present a good moisture resistance performance. Therefore, the SBS content should be seriously selected by considering the dry and wet conditions that are used to balance the adhesion property and debonding properties. The content of 4 wt.% may be the optimal content under the dry adhesion and moisture resistance.

## 1. Introduction

Asphalt mixtures as complex multi-phase materials are mainly composed of binder, aggregate, and interface phases. The interface phase is one of the weakest phases, which is prone to damage and induces several types of pavement distress, including raveling, pothole, and fatigue cracking, under repeated traffic load and environmental effects [1,2,3]. The service life of the pavement is heavily shortened by these accumulated distresses caused by the weak interface phases. In order to gain better engineering performances, good adhesion strength between asphalt binder and aggregate is required to maintain the asphalt pavement structure as a stable system [4,5]. Asphalt, as the binder material, plays the dominant role in the adhesion process with aggregates [6]. Hence, most of the studies were conducted on the modified asphalt binder to improve its adhesion property and long-term road performance [7,8,9].

Styrene–butadiene–styrene (SBS), as a favorable modifier, has been verified and widely utilized in the construction of asphalt pavement to improve the performance properties of bituminous materials over a wide range of servicing temperatures [10]. Some studies validated that the addition of proper content SBS modifiers with asphalt binder could remarkably increase the viscosity of the asphalt binder and further improve the adhesion property [11]. However, most investigations focused on engineering performance using experimental methods, while the improvement mechanism is difficult to be revealed [12,13]. Therefore, it is vital to improve the adhesion property of asphalt binder purposefully when the mix design methods are gradually changed to the performance orientation.

The molecular dynamics (MD) simulation method, as an effective method, was introduced into the materials field to reveal the micro mechanism of the formation of macro behavior at the molecular level [13,14,15]. Different mechanical properties, interaction behaviors, diffusion behaviors, and effects of the aging process, together with the moisture effect, were modeled and evaluated properly [16,17,18]. The modification mechanism was favorably illustrated by using different parameters. Moreover, an important parameter, the work of adhesion, was proposed to characterize the adhesion behavior between asphalt binder and aggregate [17]. However, most MD-related investigations mainly focused on the effect of unmodified and aged binders on the adhesion between binder and aggregates; only limited studies considered the effect of the SBS modifier [19,20,21].

The objective of this study was to investigate the interface behavior between SBS-modified asphalt binder and aggregates, where the adhesion property and moisture effect were focused on revealing their interface adhesion mechanism. Firstly, the representative models of asphalt binder, styrene–butadiene–styrene (SBS) modifier, and aggregate were built based on molecular dynamics software Materials Studio 2021. Next, the double-layer model of SBS modified asphalt binder–aggregate with different SBS contents was constructed to evaluate the adhesion property. The geometry optimization and dynamics equilibration were conducted afterward, followed by the calculation of work of adhesion to better understand the influence of SBS content. Then, the triple-layer model of asphalt binder–water–aggregate was constructed, and the moisture effect was further analyzed by adding the 100 water molecules (about 10 wt.%) in the interlayer of asphalt binder–aggregate. Finally, the work of debonding and energy ratio was selected as the evaluation parameters to evaluate the moisture effect. The obtained result can be used for the selection of SBS modifier content and to understand the interface behavior.

## 2. Materials and Methods

### 2.1. Materials

**Figure 1:**
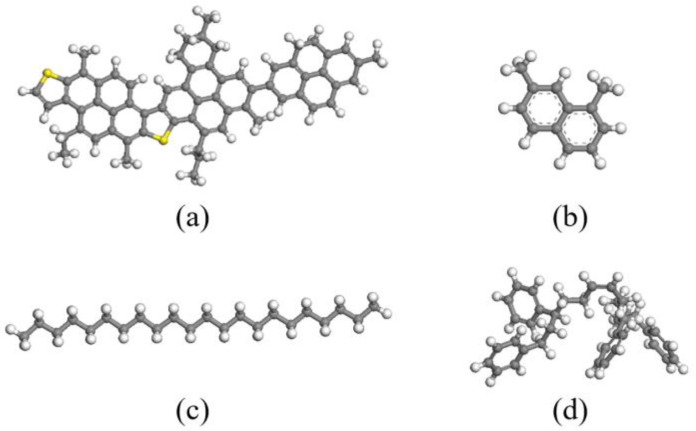
Molecular structures of SBS modified asphalt compositions for molecular simulations: (**a**) asphaltene; (**b**) naphthene aromatic; (**c**) saturate; (**d**) SBS.

In this study, the interface behavior between SBS-modified asphalt binder and aggregates was investigated. Therefore, the types of asphalt binder, SBS modifier, and aggregates should be selected. Asphalt binder as a mix consists of thousands of compounds [22]. Different models, such as the average molecular model and components constructed model, were used to represent the asphalt binder [23,24,25,26]. The average mode was proposed using Nuclear Magnetic Resonance (NMR) spectroscope, which can simulate asphalt binder accurately but fail to describe the fraction differences in asphalt binder. Multiple component models were firstly proposed by Zhang and Greenfield in 2007 to represent asphalt binders more accurately [27]. The components model was verified and widely used after it was developed [28,29]. In this study, the three-component constructed model was selected, as shown in Figure 1a–c, where 1,7-dimethylnapthalene and n-docosane (n-C_22_H_46_) were used to represent naphthene aromatic and saturate, respectively. The weight proportion of asphaltenes, naphthene aromatics, and saturates is approximately 20:20:60, respectively; the corresponding molecular number is 5, 27, and 41, respectively. The reliability of molecular structures and numbers has been verified by plenty of researchers [28,29].

After the constituent molecules of the asphalt binder were determined, the SBS molecular structure was then selected. In this study, a linear SBS structure with a molecular weight of 471 was selected (Figure 1d), where the weight proportion was nearly 2% by the weight of the asphalt binder.

Various types of aggregates, including basalt, granite, quartzite, limestone, etc., were used for asphalt pavement construction. Among them, quartz is the most common mineral with high proportions in mineral compositions [30]. Thus, quartz was selected in this study as the representative of aggregate.

### 2.2. Modeling of Asphalt Binder–Aggregate Interface

As for the adhesion simulation, the double-layer model of asphalt binder–aggregate needed to be built. The single molecular models of asphalt binder and aggregate had to be built separately. The asphalt binder model was built by assembling three components together with the corresponding molecular numbers. Similarly, the models of SBS modified asphalt binder with different SBS contents (0 wt.%, 2 wt.%, 4 wt.%, 6 wt.%, 8 wt.%, 10 wt.%) were built by assembling asphalt binder components with SBS structure of 1, 2, 3, 4, and 5, respectively.

As for the aggregate, the α-quartz was imported from the software database, and then the (1,1,0) plane was cleaved to obtain a quartz surface. Afterward, the two-dimensional (2D) structure of the quartz surface was created by increasing the unit cell to 4 and 6 for U and V directions, respectively. Then, a three-dimensional quartz crystal was obtained by adding a vacuum slab with a thickness of zero. Moreover, the hydroxylation of the surface silica was also considered.

**Figure 2:**
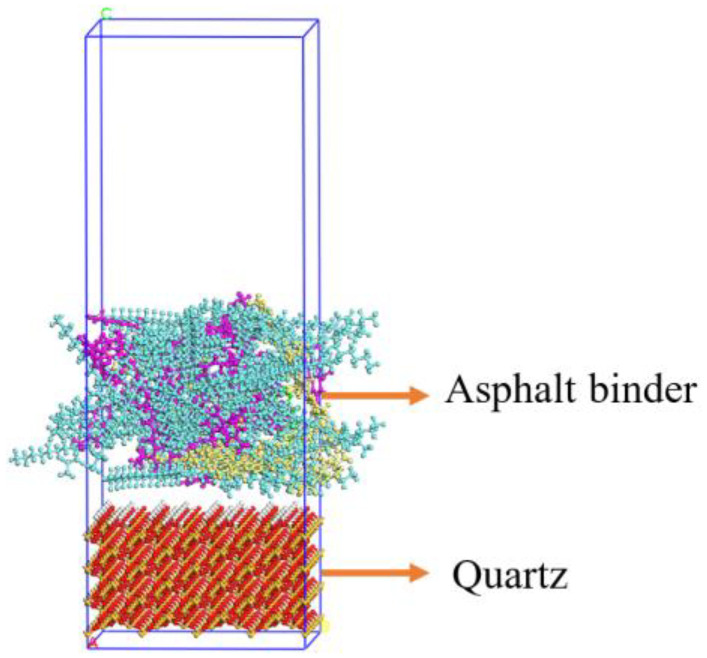
Double-layer interface model of SBS modified asphalt binder–aggregate.

After the confined SBS modified asphalt binder model and aggregate model were built, the asphalt binder–aggregate interface model could be constructed, as shown in Figure 2 (taking the interface model with 2 wt.% SBS modifiers as an example).

### 2.3. Modeling of Asphalt Binder–Water–Aggregate Interface

**Figure 3:**
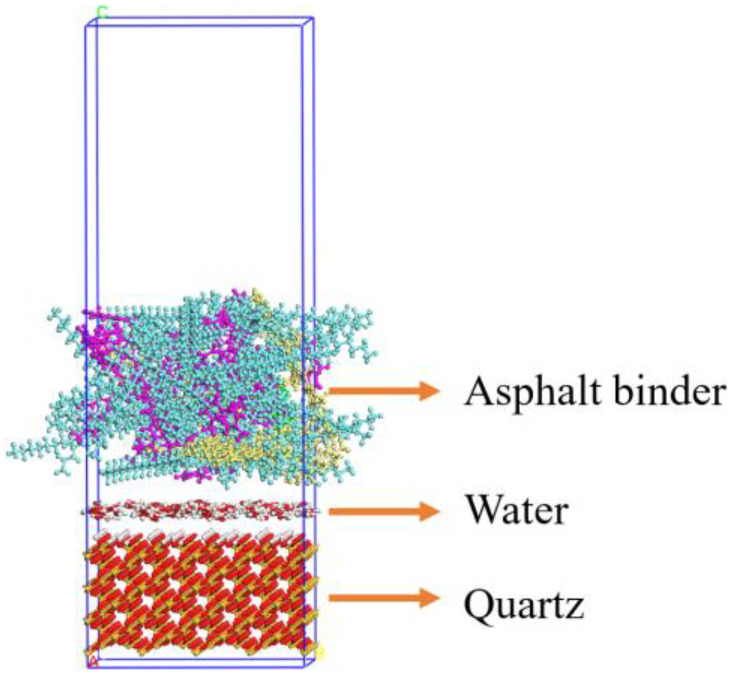
Triple-layer interface model of SBS modified asphalt binder–water–aggregate.

Water molecules of 100 were added to the interface between the SBS-modified asphalt binder layer and aggregate layer to characterize the moisture effect of adhesion, where the weight proportion was nearly 10 % of asphalt binder by weight. The built triple-layer interface model for asphalt binder–water–aggregate was shown in Figure 3 (taking the layer model with 2 wt.% SBS modified asphalt–aggregate–quartz as an example).

### 2.4. Simulation Details and Evaluation Index

All MD simulations were performed using Materials Studio 2021 with the COMPASS II force field to represent the atomistic interactions. COMPASS II force field is optimized ab initio force field and can be used to accurately simulate organic, inorganic, and polymer material [31]. The total potential energy (E_total_) consists of valence and nonbonded interaction terms. The valence term (E_val_) includes the interactions of bond stretching (E_b_), angle bending ($E_{\theta }$), internal torsion ($E_{\varphi }$), out-of-plane bending ($E_{\chi }$), and the cross-coupling terms (E_bb′_, $E_{b\theta }$, $E_{b\varphi }$, $E_{{\theta \varphi }^{\prime }}$, $E_{{\theta \theta }^{\prime }}$, and $E_{{\theta \theta }^{\prime }\varphi }$). The non-bond interaction term (E_non-bond_) quantifies the non-covalent contributions, including Coulomb electrostatic energy (E_elec_) and the van der Waals energy (E_LJ_) [32].

After all constituent molecules of asphalt binder and SBS structure were determined. The bulk models of SBS modified asphalt binder with different SBS contents were built. The bulk model is an amorphous cell with a three-dimensional periodic boundary. The geometry optimization with 10,000 iterations was conducted afterward to eliminate the highest energy overlap. The optimized configuration was further refined by performing Forcite dynamic calculations using Material Studio 2021 under the isothermal–isobaric (NPT) ensemble for 500 ps and 1 atm pressure and the canonical ensemble (NVT ensemble) for 500 ps to ensure that the system reached an equilibrium state. The temperature was set as 298 K, which is a common service temperature and has been widely used in this previous adhesion-related simulations [32,33]. The Ewald and the atom-based summation methods were used for electrostatic interactions and the Van der Waals interactions. The cutoff distance was set as 15.5 Å (this parameter was selected by considering the minimum image convention, the simulation cost, and the previous studies together).

After the bulk models were fully equilibrated, the density of SBS-modified asphalt binders was calculated. The experimental results were used to validate the built model’s simulation reliability. The confined models were then further constructed to build the double-layer models. Different from the bulk model, the cell boundary for the confined model in the z-direction was confined by the hard repulsive wall, which stops any movements in the box beyond the wall. The confined single-layer models for the SBS modified asphalt binder were also optimized and equilibrated using the same procedures as the bulk one.

The double-layer models were then built to add the confined asphalt binder models to the top of the aggregate supercell. A vacuum of 40 Å was added above the top aggregate layer to avoid the interaction across the mirror image in the z-direction. The geometry optimization with 5000 iterations was conducted afterward for the built double-layer models, followed by the dynamic equilibration under the canonical ensemble (NVT ensemble) for 300 ps. It should be noted that the bottom aggregate layer was fixed during the optimization and dynamics simulation. The obtained stable configuration was selected for further adhesion strength calculation.

As previously stated, the work of adhesion, as a thermodynamic parameter, was selected to characterize the adhesion property between SBS-modified asphalt binder and aggregate [17]. This parameter is defined as the required energy to separate a unit area of an interface into two free surfaces in a vacuum. A better adhesion performance was presented with a larger work of adhesion. The work of adhesion was calculated by Equation (1) for the asphalt binder–aggregate layer model [34]. Specifically, the total potential energy of the asphalt binder–aggregate system under the equilibration state was calculated first. The potential energy for asphalt binder and aggregate could then be obtained by deleting the asphalt binder layer and aggregate layer from the system, respectively. Finally, the work of adhesion can be obtained using Equation (1).

(1) $$W_{adhesion}=(\left(E_{a}{+E}_{agg}\right)-E_{total})/A,$$

where:

$W_{adhesion}$ is the work of adhesion between asphalt binder and aggregate;
*E_total_* is the total potential energy of asphalt binder–aggregate interface model;
*E_a_* is the potential energy of the asphalt binder;
*E_agg_* is the potential energy of the aggregate;
*A* is the interface contact area between the asphalt binder and aggregate surface.

For the moisture effect, the optimization and dynamics simulation process for the asphalt binder–water–aggregate model was the same as the asphalt–aggregate model. After the stable configuration was obtained, the work of debonding for water to displace asphalt binder from its interface with aggregate was calculated according to Equation (2) [32]. Good moisture resistance was provided with the small work of debonding. Similar to the calculation procedures of the work of adhesion, the work of debonding was calculated after the triple-layer system reached the equilibration state. The interaction energy between two materials was calculated by deleting other materials.

(2) $$W_{debonding}=\left(\left(\Delta E_{a\_w}+\Delta E_{agg\_w}\right)-\Delta E_{a\_agg}\right)/A,$$

where:

*W_debonding_* is the work of debonding;
Δ*E_a_w _* is the interaction energy between asphalt and water;
Δ*E_agg_w_* is the interaction energy between aggregate and water;
Δ*E_a_agg_* is the interaction energy between aggregate and asphalt.

Another parameter, energy ratio (*ER*), was also proposed to characterize the moisture resistance comprehensively [35]. *ER* is calculated by the ratio of the work of adhesion to the work of debonding, as shown in Equation (3). A higher *ER* value means better moisture resistance, which indicates better adhesion and low moisture damage susceptibility.

(3) $$ER=W_{adhesion}/W_{debonding},$$

## 3. Results and Discussion

### 3.1. Verification of the Built MD Models

**Figure 4:**
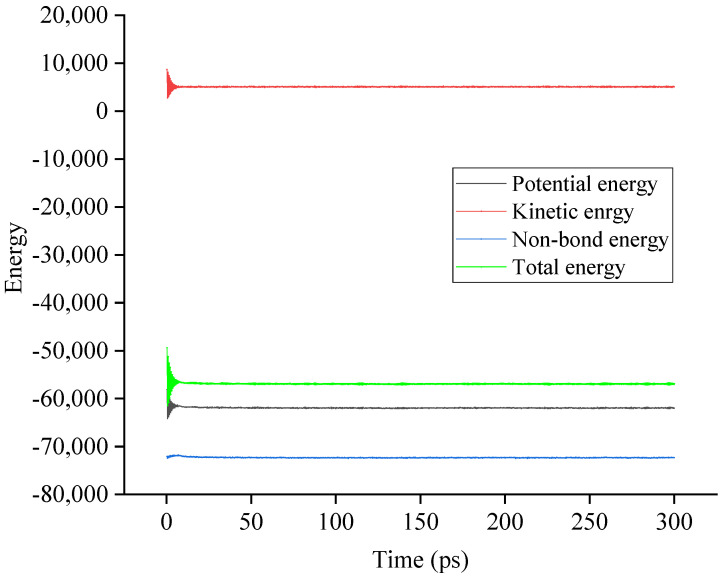
Variation in energy during the NVT dynamics equilibration.

**Figure 5:**
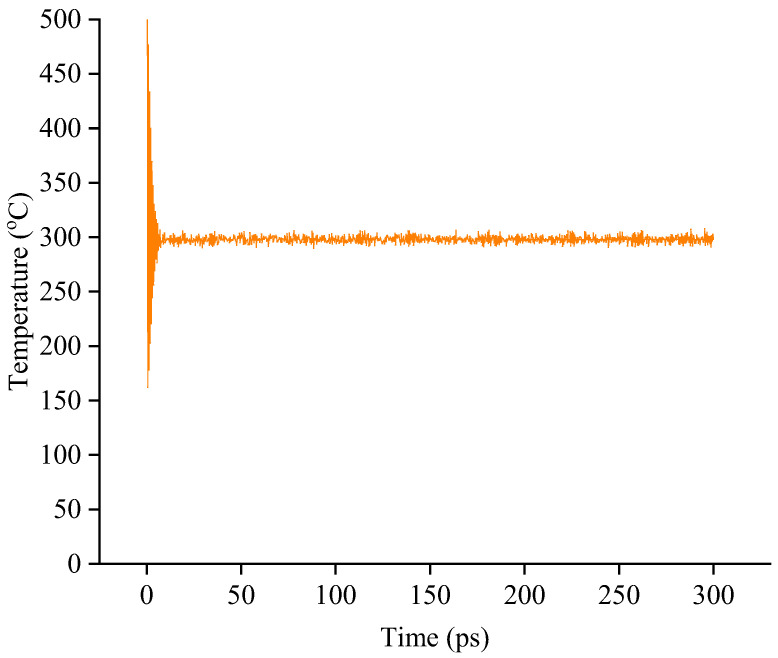
Variation in temperature during the NVT dynamics equilibration.

The molecular dynamics (MD) simulation was conducted using the detailed procedures in Section 2.3. The energy and temperature were monitored during the dynamic equilibration process for the single-layer confined SBS modified asphalt binder models, double-layer interface models, and triple-layer interface models. Considering that the simulation was conducted under the NVT ensemble, the system should have constant total energy and temperature under the equilibration state. Thus, whether the system reaches the equilibration state can be judged by these two parameters. By taking SBS-modified asphalt binder–aggregate with 2 wt.% SBS content as the example, the variations in energy and temperature during the NVT dynamics equilibration are shown in Figure 4 and Figure 5.

As shown in Figure 4 and Figure 5, the energy and temperature fluctuated within an initial time of 10 ps while remaining at a relatively constant value with the increase in time. The constant value means that the built models are fully optimized and reach the equilibration state under the NVT ensemble. As for all single-layer confined SBS modified asphalt binder models, double-layer interface models, and triple-layer interface models, similar results are obtained, which verifies that the built models are under the equilibration state and can be used for further adhesion and debonding properties analysis.

After the models were fully optimized and equilibrated, the density of the SBS-modified asphalt binder under 298 K was calculated and compared with the previous studies to verify the reliability of the built model [36]. It should be noted that the SBS content of 0% means the unmodified asphalt binder, and its density was compared with the previous work, while the density for other SBS modified asphalt binder was estimated by the density of SBS modifier and asphalt binder using the hypothesis of volume additivity.

**Figure 6:**
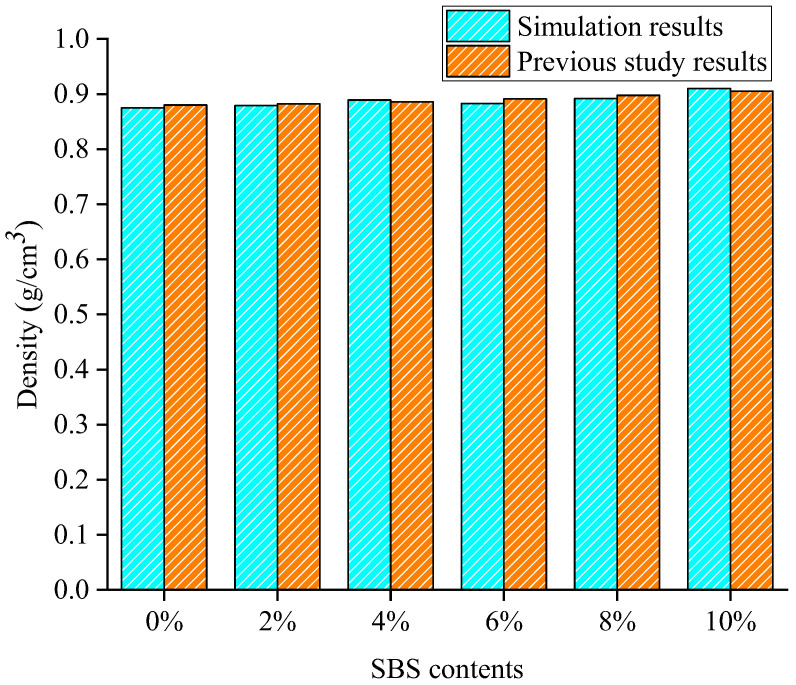
Density of SBS modified asphalt models from the simulation and previous study.

As shown in Figure 6, the density of SBS modified asphalt binder with different SBS contents from the simulation results shows good agreement with the previous study results with a maximum relative difference of 1%. Moreover, the addition of SBS into asphalt binder induces an increase in the density of asphalt binder from the simulation results, which is also consistent with the previous works [36]. This may be caused by the relatively larger density of SBS compared to the asphalt binder, with a density of 0.886. In addition, the molecular structure of SBS is more complex with the several benzene ring structures than the light components (naphthene aromatic and saturate) in asphalt binder with a large proportion. Therefore, the built SBS modified asphalt binder models reach the equilibration state and have the favorable reliability to represent the real one; thus, the models can be used for the further analysis of adhesion property and moisture effect.

### 3.2. Variation in Work of Adhesion with the SBS Contents

**Figure 7:**
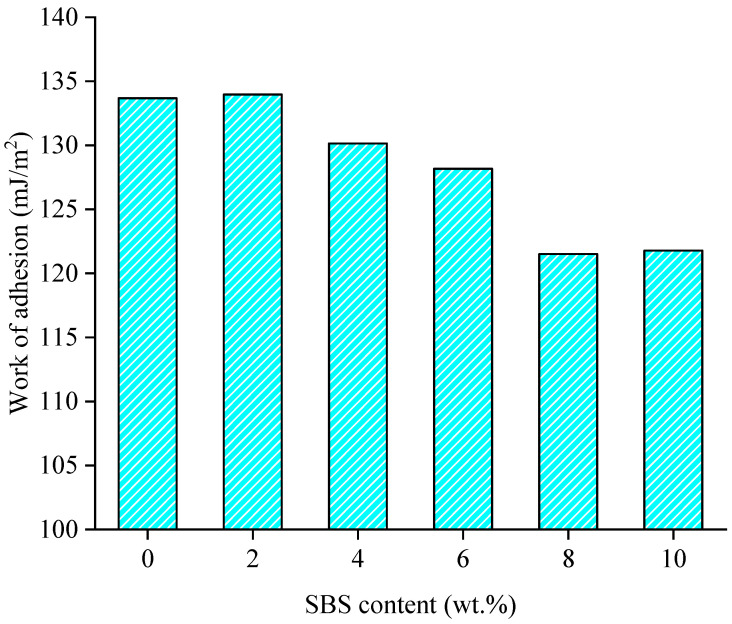
Variation in work of adhesion between SBS modified asphalt binder and quartz with SBS content.

For SBS modified asphalt binder with different SBS contents (0 wt.%, 2 wt.%, 4 wt.%, 6 wt.%, 8 wt.%, 10 wt.%), the work of adhesion between modified asphalt and aggregate can be calculated according to Equation (1). The adhesion results are shown in Figure 7.

As can be seen from Figure 7, the variation in the work of adhesion for SBS modified asphalt binder–quartz is not visible with the SBS content when its content is lower than 6 wt.%. However, the work of adhesion decreases significantly relatively when the content is high, which is consistent with the experimental results [36]. The former may attribute to the addition of SBS increased the benzene ring structure and cross-linked with asphalt binder to form a stable network structure, inducing the increase in adhesion within the small amount of SBS contents. The latter may be caused by poor compatibility when the content is relatively large (8% and 10 %), and the poor compatibility may result in a decrease in the adhesion property. Moreover, the work of adhesion between SBS modified asphalt binder and quartz is derived from the Van der Waals energy from the energy compositions, where the contribution of Van der Waals energy is minor and limited from the previous works [21]. Therefore, the variation in the work of adhesion is not remarkable.

### 3.3. Moisture Effect on the Interface of SBS Modified Asphalt Binder and Aggregate

**Figure 8:**
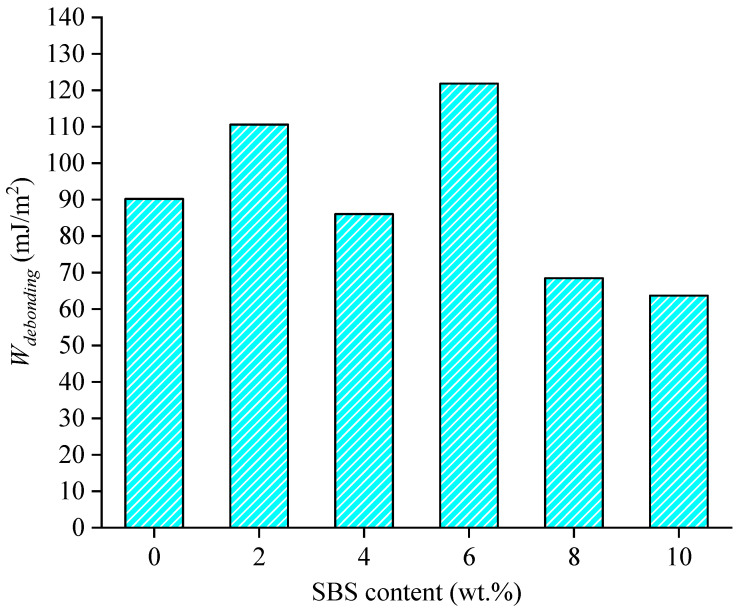
Variation in work of debonding with SBS content.

**Figure 9:**
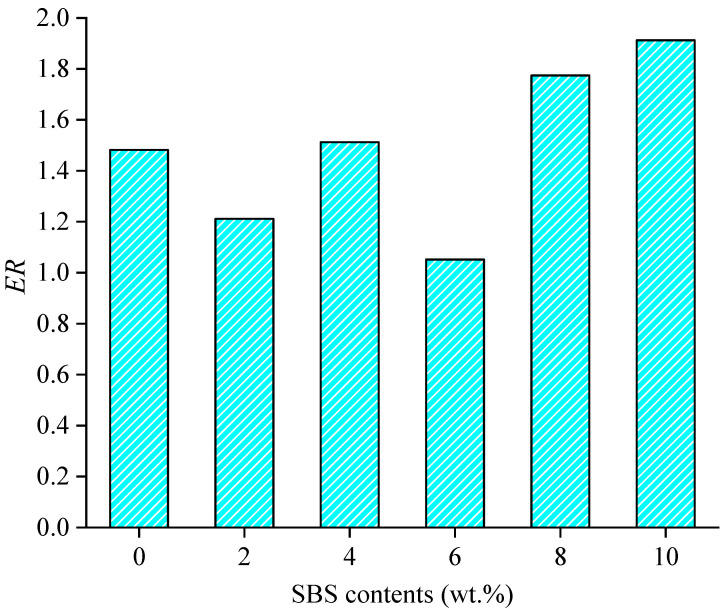
Variation in *ER* values with SBS content.

The moisture effect was analyzed by adding water molecules into the interface of asphalt–aggregate. The same SBS contents were used to analyze the effect of water on SBS-modified asphalt binder–aggregate with different SBS contents. The work of debonding is calculated as shown in Figure 8. The *ER* values are calculated as well as shown in Figure 9.

As can be seen from Figure 5, the moisture effect for adhesion properties under different SBS contents is significant, especially for the asphalt binder–water–aggregate model with 6 wt.% SBS modifiers. The work of debonding for SBS modified asphalt binder–quartz increases firstly and decreases afterward with the SBS content except for the one with 4 wt.% SBS modifiers. This may be because the addition of SBS modifiers with the small percentage content into asphalt binder may increase the interaction energy of asphalt binder with water while decreasing the interaction energy of asphalt binder with the higher content. The larger interaction energy represents the strong interaction and the good affinity. Moreover, the modified asphalt binder with a high content (8 wt.% and 10 wt.%) of SBS modifiers shows much better moisture resistance than the unmodified asphalt binder, where the relative reduction ratio of work of working is about 30%. This means the addition of an SBS modifier has a positive effect on moisture resistance. Therefore, the asphalt binder with a high content of SBS modifiers may be more favorable from the debonding aspect.

In order to comprehensively characterize the interface performance between SBS modified asphalt binder and aggregate, the *ER* values were applied. As shown in Figure 9, the *ER* values for all asphalt binder–aggregate models are larger than 1, which verifies that all modified asphalt binder has good moisture resistance. The asphalt binder with high content SBS presents a good performance in interface property for the higher *ER* values. This is mainly caused by the small value of the work of debonding. However, the large content of SBS modifiers means a high cost. The SBS content should be selected by considering the environment that is used to balance the adhesion property and debonding property.

## 4. Conclusions

In this study, the adhesion property and moisture effect between SBS-modified asphalt and aggregate were investigated using molecular dynamics simulation. The corresponding double-layer adhesion models and triple-layer debonding models were built. The variation in adhesion and debonding properties with SBS contents were investigated. The following conclusions were obtained:

(1) The built SBS modified asphalt binder models were verified to reach the equilibration state and have the favorable reliability to represent the real one from the dynamics simulation process parameters, e.g., energy and temperature, and density of SBS modified asphalt binder, thus, the models can be used for the further analysis of adhesion property and moisture effect;

(2) The variation in the work of adhesion for SBS modified asphalt binder–quartz is insignificant with the SBS content. The work of adhesion for SBS modified asphalt binder–quartz can be derived from the Van der Waals energy;

(3) The presence of water significantly reduces the interface adhesion. The modified asphalt binder with a high content (8 wt.% and 10 wt.%) of SBS modifiers shows much better moisture resistance than the unmodified asphalt binder from the results of work of debonding;

(4) The asphalt binder with high content SBS presents a good performance in moisture resistance according to *ER* values. Moreover, the SBS content should be selected by considering the environment that is used to balance the adhesion property and debonding property. The content of 4 wt.% may be the optimal content under the dry adhesion and moisture resistance.

This study used quartz to present the aggregate, and linear SBS modifiers represented SBS. Further studies should be conducted to investigate the impact of different types of aggregates and SBS modifiers on adhesion and debonding properties. Moreover, the experimental methods using AFM and surface free energy theory should be conducted to further reveal the modification mechanism and performance variations.

## Data Availability

Not applicable.
